# Extension of the Medical Session in Our University

**Published:** 1848-03

**Authors:** 


EXTENSION OF THE MEDICAL SESSION IN OUR UNIVERSITY.

The annual advertisement of the Faculty, appended to the Catalogue of Students just issued by the Dean, announces that the next session will commence on the 16th day of October; which being Monday, and occurring just three weeks before the first Monday of November, (the 6th day) will make the next session twenty weeks instead of seventeen. We understand that it is the intention of the Faculty to commence the session, following the next, on the first Monday of October, thus adding a month to the old term. We record this attempt with heart felt pleasure, for our short sessions, in the United States, may justly be considered, as adverse to adequate preparation, for graduation.

